# Case report and literature review of rezvilutamide in the treatment of hormone-sensitive prostate cancer

**DOI:** 10.3389/fonc.2024.1374039

**Published:** 2024-03-21

**Authors:** Chunlei Zhang, Jie Ren, Yindong Kang, Dehui Chang

**Affiliations:** Department of Urology, The 940 Hospital of Joint Logistics Support Force of Chinese PLA, Lanzhou, Gansu, China

**Keywords:** prostate cancer, rezvilutamide, metastatic hormone-sensitive prostate cancer (mHSPC), androgen deprivation therapy (ADT), genetic testing

## Abstract

**Background:**

Prostate cancer represents a major health concern worldwide, with the treatment of metastatic hormone-sensitive prostate cancer (mHSPC) and locally advanced prostate cancer posing a particular challenge. Rezvilutamide, a new androgen receptor antagonist from China, has shown early promise; however, its real-world effectiveness and safety profile require further evidence. This case series evaluates the preliminary clinical outcomes of rezvilutamide in combination with androgen deprivation therapy (ADT), focusing on PSA response and radiological findings across various stages of prostate cancer in four patients.

**Case description:**

Case 1 details a 68-year-old male with low-volume mHSPC who exhibited a positive therapeutic response, demonstrated by decreasing PSA levels and improved radiographic results, despite experiencing mild side effects related to the drug. Case 2 describes a 71-year-old male with high-volume mHSPC who had a favorable outcome, with no significant changes in tumor size or metastatic spread and no negative reactions to the drug. Case 3 involves a 55-year-old male with locally advanced prostate cancer, who saw a reduction in PSA levels and a small decrease in tumor volume, yet with ongoing bladder involvement. Genetic testing showed no significant mutations. Case 4 presents a 74-year-old male with extensive metastatic disease who initially responded to the treatment but later exhibited disease advancement and an *ATM* gene mutation, signaling a shift to metastatic castration-resistant prostate cancer (mCRPC). This finding underscores the crucial role of genetic testing in directing future treatment, with therapies such as olaparib or chemotherapy being advised.

**Conclusions:**

Rezvilutamide has shown a potential benefit in the management of mHSPC and locally advanced prostate cancer, generally with a mild safety profile. Initial positive responses, particularly in PSA decline and radiographic progression, are promising. Nevertheless, the varying responses, notably concerning genetic mutations, highlight the necessity for tailored treatment approaches. Due to the small cohort and brief follow-up period, more extensive research with larger populations and prolonged monitoring is essential to conclusively determine the benefits and safety of rezvilutamide. The utilization of genetic insights is key to refining treatment decisions and enhancing outcomes for patients with advanced prostate cancer.

## 1 Introduction

The management of prostate cancer has made considerable progress, especially in developing treatments for metastatic hormone-sensitive prostate cancer (mHSPC), which have seen substantial evolution in recent times. Rezvilutamide, an innovative androgen receptor antagonist, marks a significant milestone as it is the first drug of its kind to be developed domestically in China through original research (1). Its potential as a therapeutic agent has been recognized in preliminary studies. Despite this, there is a notable gap in real-world clinical data regarding its effectiveness and safety. This deficit calls for additional validation via clinical practice observations. The present case series aims to fill this gap by documenting the PSA response and radiographic results in four prostate cancer patients who received rezvilutamide alongside androgen deprivation therapy (ADT). This narrative serves to illustrate the initial clinical performance of rezvilutamide.

## 2 Case report

**Figure 1:**
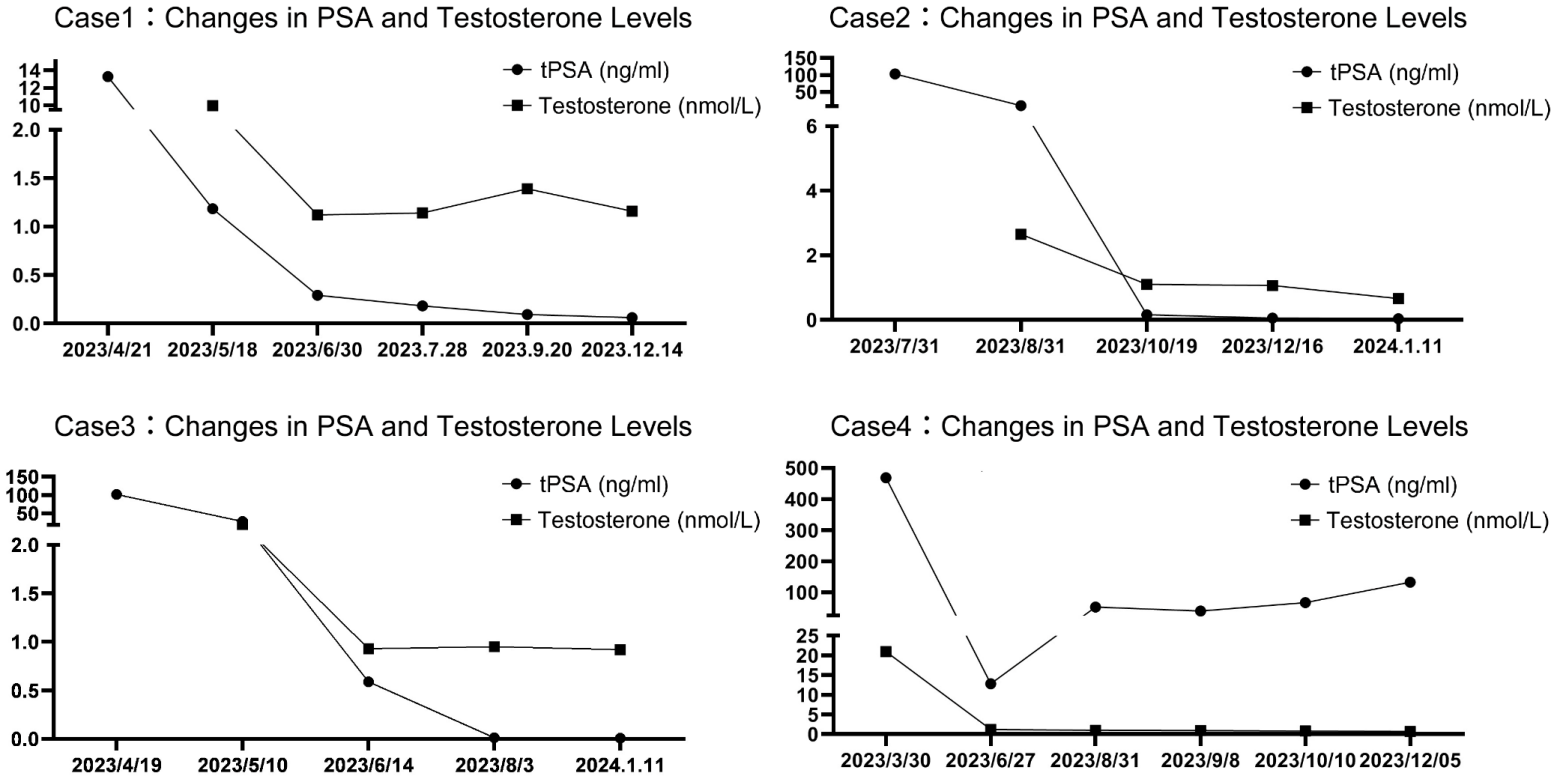
The changing trends of PSA and testosterone levels over time in four patients.

**Case 1**: A 68-year-old man with five years of progressive urination difficulty, but no other symptoms, chronic illnesses, or family history of cancer or genetic disorders, was admitted. Initial tests revealed high PSA levels (13.280 ng/ml total PSA (tPSA), 0.29 free PSA (fPSA)/tPSA ratio). MRI suggested prostate cancer with possible bladder and seminal vesicle invasion, a prostatic abscess, and suspected metastases in the sacral, retroperitoneal, and lymphatic regions. Bone scans indicated sacral and sacroiliac joint metastases. A 12 standard and 3 targeted cores biopsy on April 18, 2023, confirmed cancer in 8 of 15 cores with a Gleason score of 8 (ISUP group 4).He was diagnosed with stage cT4NxM1b prostate cancer and treated with leuprorelin acetate (3.75 mg every 28 days) plus daily rezvilutamide (240 mg). Nine months later, testosterone remained low, PSA decreased, as shown in **Figure 1**; **Table 1**. Follow-up MRI and bone scans showed reduced prostate volume and metastases. He experienced transient mild eyelid edema and blurred vision, which resolved without halting the treatment. Liver and kidney function stayed normal.

**Table 1:** Characters of series cases.

Case	Age	TNM	Disease stage	Therapy	PSA changes (ng/ml)	Testosterone changes (nmol/L)	Efficacy	Gene test
1	68	cT4NxM1b	mHSPC (Low-volume)	Leuprorelin acetate + Rezvilutamide	13.280→0.061	9.98→1.16	PR	–
2	71	cT4N1M1b	mHSPC (high-volume)	Goserelin Acetate + Rezvilutamide	103.256→0.037	2.65→0.66	SD	–
3	55	cT3-4NxMx	Localized advanced	Goserelin Acetate+ Rezvilutamide	101.338→0.009	18.98→0.92	PR	Negtive
4	74	cT2cN1M1c	mHSPC (high-volume)→mCRPC	Goserelin Acetate + Rezvilutamide	468.886→12.8→133.297	20.95→0.73	PR→PD	*ATM* mutation

mHSPC, metastatic Hormone-Sensitive Prostate Cancer; PR, partial response; SD, stable disease; PD, progress disease. “→” means changes in PSA and testosterone. "-" means no genetic testing was performed.

**Case 2**: A 71-year-old man with a five-year history of worsening urination and urolithiasis, but no cancer or genetic disorders, was hospitalized. Despite these issues, his diet, bowel habits, and weight were stable, yet his PSA was alarmingly high (tPSA at 103.256 ng/ml and a fPSA/tPSA ratio of 0.36). Pelvic MRI and bone scans revealed widespread prostate cancer with bladder and seminal vesicle involvement, bone metastases including the sacrum, L5 vertebra, and hips, and right iliac lymph node involvement. On July 25, 2023, the patient had a transperineal prostate biopsy with 12 standard and 3 targeted cores, and a transurethral bladder tumor resection, under spinal anesthesia. Pathology confirmed prostate cancer; 10 of the 15 positive cores had Gleason scores of 9 or 10, classified as aggressive ISUP group 5, two scored 8 (group 4), and three scored 7 (group 3), with perineural invasion noted in four cores. Diagnosed with stage cT4N1M1b prostate cancer, he received goserelin acetate injections (3.6 mg every 28 days) and daily oral rezvilutamide (240 mg). Over six months, his testosterone dropped to castration levels, PSA levels fell, and imaging indicated stable disease with no drug adverse reactions, as **Figure 1**; **Table 1** shows. Liver, kidney, and electrolyte levels remained within normal range.

**Case 3:** A 55-year-old male with 18 months of urination difficulty and 3 days of severe hematuria had no other symptoms, maintained normal eating and bowel habits, and stable weight. He had no previous diseases, cancers, or family genetic disorders. Elevated PSA levels (101.338 ng/ml tPSA, 0.12 fPSA/tPSA ratio) and pelvic MRI indicated prostate cancer, with unclear borders near the bladder, seminal vesicles, lymphadenopathy near iliac vessels and inguinal area, and sacral anomalies. Bone scintigraphy showed no bone metastases. On April 13, 2023, he had a 12-core plus 3-core targeted transperineal prostate biopsy under spinal anesthesia, revealing prostate cancer with 14 positive cores, including 7 cores with a Gleason score of 9 (ISUP group 5), 4 cores scored 8 (group 4), and 3 cores scored 7 (group 3). He was diagnosed with stage cT3-4NxMx prostate cancer. Genetic testing did not identify clinically significant gene mutations. Treatment included goserelin acetate injections (10.8 mg every three months) and daily oral rezvilutamide (240 mg). Over nine months, his testosterone remained at castrate levels, PSA levels fell gradually, as **Figure 1**; **Table 1** shows. After six months, he declined radical surgery or radiotherapy. Follow-up MRI showed slight lesion improvement, persistent bladder and seminal vesicle invasion, unchanged lymphadenopathy, and sacral signal, with a normal bone scan. No side effects were reported, and his liver, kidney, and electrolyte tests stayed normal.

**Case 4**: A 74-year-old male with a two-year history of worsening urinary difficulty, hyperhidrosis, fatigue, myalgia, and poor appetite but stable weight was admitted. He had a history of a left leg fracture repair, a 50-year smoking habit, but no significant diseases, cancers, or family genetic disorders. His PSA levels were very high (tPSA 468.886 ng/ml, fPSA/tPSA ratio 0.13). CT scans revealed prostate cancer with lung and pleural metastases, and bone scintigraphy showed extensive metastatic lesions with increased radiotracer uptake in the skull, sternum, bilateral scapulae and clavicles, multiple ribs, vertebral bodies, pelvic bones, and long bones of the limbs. A prostate biopsy on March 23, 2023, confirmed prostate cancer with varied Gleason scores across all 12 cores The Gleason scores were distributed as follows: 1 core scored 6 (group 1); 5 cores scored 7 (group 2); 2 cores scored 7 (group 3); 2 cores scored 8 (group 4); and 2 cores scored 9 (group 5). The diagnosis was prostate cancer staged as cT2cN1M1c. Post-discharge, he received goserelin acetate (3.6 mg every 28 days) and daily rezvilutamide (240 mg), maintaining castration-level testosterone. By June 27, PSA levels dropped to 12.8 ng/ml, with improved bone metastases. However, PSA rose to 133.297 ng/ml by December 5 (Shown in **Figure 1**), suggesting metastatic castration-resistant prostate cancer (mCRPC). An *ATM* gene mutation was found by genetic testing, and treatment plans considered olaparib or chemotherapy. He had transient mild facial and hand edema, which resolved without stopping treatment. These findings are summarized in **Table 1**.

## 3 Discussion

Prostate cancer is a prevalent cancer in men worldwide, with early-stage patients often having a good prognosis, unlike those with advanced or metastatic disease, who fare worse, especially in less developed areas with higher rates of advanced presentations (2). The standard treatment, especially for advanced cases, is androgen deprivation therapy plus antiandrogens. While first-generation antiandrogens like bicalutamide and flutamide have been beneficial, newer antiandrogens such as abiraterone, enzalutamide, apalutamide, and darolutamide more effectively manage prostate cancer, enhancing life quality and survival chances (3). Rezvilutamide (SHR3680), a new Chinese androgen receptor inhibitor, has been clinically approved. It provides similar benefits to earlier treatments, with fewer risks than abiraterone and prednisone, which may cause hepatotoxicity, blood sugar issues, and osteoporosis, and less risk of seizures or other side effects like fatigue and rash associated with enzalutamide and apalutamide, all while being simpler to administer.

Recent phase III trials have confirmed the benefits of new endocrine treatments for mHSPC. The LATITUDE study showed adding abiraterone and prednisone to ADT significantly raised overall survival (OS) over androgen deprivation therapy (ADT) alone, notably for those with visceral metastases (4, 5). The TITAN trial reported that ADT with apalutamide lengthened OS more than ADT alone, with less impact on patients with visceral metastases (6). ENZAMET and ARCHES trials found that ADT plus enzalutamide improved outcomes similar to TITAN’s findings (7, 8). The ARASENS trial indicated that ADT, docetaxel, and darolutamide extended OS in mHSPC patients, notably for visceral metastases, compared to ADT and docetaxel (9). CHART study results with rezvilutamide paralleled these findings, showing significant OS improvement in mHSPC, though less for patients with visceral metastases (10). **Table 2** showcases these trials’ comparative data. In this cases analysis, rezvilutamide demonstrated efficacy in low-volume mHSPC (case 3), high-volume mHSPC (case 2 and case 4), and locally advanced prostate cancer (case 1). After several months of treatment, all patients achieved effective disease control in the short term, although in case 4, the patient experienced disease progression after 5 months of therapy.

**Table 2:** Phase III Clinical Trial of Novel Endocrine Therapy for mHSPC.

No.	Trial name	Treatment	Results	Ref.
1	LATITUDE	ADT + abiraterone + prednisone (N = 597) vs ADT + placebo (N = 602)	Median OS: 53.3 months vs 36.5 months (HR 0.66; 95% CI 0.56-0.78; P < 0.001); Median PFS: 33.0 months vs 14.8 months (HR of progression or death 0.47; 95% CI 0.39-0.55; P < 0.001)	(4, 5)
2	TITAN	ADT + apalutamide (N = 525) vs ADT + placebo (N = 527)	OS at 24 months: 82.4% vs 73.5% (HR 0.67; 95% CI 0.51-0.89; P = 0.005); PFS at 24 months: 68.2% vs 47.5% (HR 0.48; 95% CI 0.39-0.60; P < 0.001)	(6)
3	ENZAMET	ADT + enzalutamide (N = 563) vs ADT + nonsteroidal antiandrogen agent (N = 562)	Median OS: NR vs 73.2 months (HR 0.70; 95% CI 0.58-0.84; P < 0.001); 5-year overall survival: 67% (0.63-0.70) vs 57% (0.53-0.61)	(7)
4	ARCHES	ADT + enzalutamide (N = 574) vs ADT + placebo (N = 576)	OS: Not mature; rPFS: NR vs 19.0 months (HR 0.39; 95% CI 0.30-0.50; P < 0.001)	(8)
5	ARASENS	ADT + darolutamide + docetaxel (N = 651) vs ADT + placebo +docetaxel (N = 655)	Median OS: NR vs 48.9 months (HR 0.68; 95% CI 0.57-0.80; P < 0.001); Median time to CRPC: NR vs 19.1 months (HR 0.36; 95% CI 0.30-0.42; P < 0.001)	(9)
6	CHART	ADT + rezvilutamide (N = 326) vs ADT + bicalutamide (N = 328)	Median OS: NR vs NR (HR 0.58; 95% CI 0.44-0.77; P < 0.001); Median rPFS: NR vs 25.1 months (HR 0.44; 95% CI 0.33-0.58; P < 0.001)	(10)

Clinical research has shown that reductions in PSA levels can forecast the outcome for metastatic prostate cancer patients. PSA kinetics, including the rate and extent of decrease, are strongly linked to cancer progression, serving as key measures for treatment monitoring and relapse assessment (11–14). A PSA level ≤ 0.2 ng/mL at 7 months after therapy predicts better OS for mHSPC patients on ADT (15). At the AMCP Nexus conference, it was reported that treatments like apalutamide, enzalutamide, or abiraterone achieved a median of 2.7 to 6.2 months to reach a 90% reduction in PSA (PSA90), and 3.5 to 9.7 months to PSA < 0.2 ng/mL (16). The decline in PSA was notably more rapid among Asians, as 73.9% reached PSA < 0.2 ng/mL within two months post-apalutamide treatment in the TITAN study (6). The CHART study found a median 29 days to PSA90 and 68.7% reached PSA < 0.2 ng/mL by week 12 (10). A multicenter trial with rezvilutamide showed a 75.7% rate of PSA response (PSA reduction ≥ 50%) by week 12 in non-chemotherapy patients (17). Substantial testosterone reduction also improves outcomes and may delay progression to CRPC (18–20). All case reports achieved PSA90 within 3 months, and except for one case, PSA levels fell below 0.2 ng/ml, with testosterone levels dropping below 1.7 nmol/L post-ADT combination.

At present, there are no standard protocols for neoadjuvant therapy or endocrine treatment prior to surgery for locally advanced prostate cancer involving new endocrine therapies. For patients with high-risk T3-T4 stage tumors, combining radical radiotherapy with endocrine or neoadjuvant therapy before surgery is an effective treatment strategy. The use of second-generation antiandrogens with ADT is becoming more common in these treatments due to new endocrine drugs (21). A meta-analysis from the STAMPEDE trial’s Phase III studies evaluated the impact of abiraterone acetate and prednisolone, with or without enzalutamide, on high-risk non-metastatic prostate cancer. Results showed that, while metastasis-free survival didn’t differ significantly, the combination therapy group saw significant boosts in OS, cancer-specific survival, biochemical recurrence-free survival, and progression-free survival compared to controls (22). Since the CHART study primarily recruited patients with a high tumor burden of mHSPC and the drug is in the early stages of clinical use, there is currently no data on the application of rezvilutamide in locally advanced prostate cancer. In case 3 of locally advanced disease, after obtaining the patient’s informed consent and with the patient’s refusal to undergo radiotherapy, we used rezvilutamide to control the disease. The patient treated with rezvilutamide and ADT experienced a minor prostate tumor shrinkage, but bladder invasion risk remained. This may provide a reference for the future application of rezvilutamide as a treatment option for locally advanced prostate cancer. Further research is needed to confirm rezvilutamide’s effectiveness as neoadjuvant therapy.

The use of next-generation sequencing (NGS) for genetic testing has become critical in managing prostate cancer, enabling personalized treatment approaches. Key mutations in genes such as *BRCA1/2*, *ATM*, and *HOXB13* play a significant role in the disease’s progression and responsiveness to therapy (23–25). It is recommended for patients with mCRPC to undergo genetic profiling to detect both germline and somatic mutations, particularly in homologous recombination repair (HRR) genes, along with microsatellite instability (MSI) and DNA mismatch repair deficiency (dMMR) status assessments (23–25). This is especially important for those with a familial risk of cancer, high-grade, locally advanced, or metastatic prostate cancer. Molecular profiling guides treatment decisions, from PARP inhibitors for those with HRR deficiencies (26) to immune checkpoint inhibitors for cases with high MSI or *CDK12* loss (27, 28). In the case 3 and case 4 presented, genetic testing revealed an *ATM* mutation in one patient, which might account for the divergent PSA response to rezvilutamide treatment (29). *ATM* is crucial for DNA repair and cell cycle control, and mutations can lead to increased cancer risk. The PROfound study showed improved OS with Olaparib for mCRPC patients with BRCA1/2 or *ATM* mutations, leading to FDA approval for Olaparib in these cases in May 2020 (30). *ATM* status is also key in deciding on platinum-based chemotherapy or immunotherapy, as they depend on the cell’s ability to repair DNA. For the patient in case 4, olaparib or chemotherapy is recommended. Overall, early genetic testing is crucial for tailoring treatment, notably with emerging drugs like rezvilutamide, and for determining suitable candidates for therapies like olaparib or platinum-based chemotherapy.

AR-V7, a variant of the Androgen Receptor (AR), enhances tumor invasiveness and metastasis by activating growth factors within tumor cells (31). This variant can change its structure to resist certain anti-tumor drugs, thereby contributing to the evolution of prostate cancer into castration-resistant stages. The presence of AR-V7 is linked not only to the advancement and spread of prostate cancer but also to a decreased sensitivity to new endocrine treatments like enzalutamide and abiraterone; this has critical consequences for choosing treatment strategies and evaluating patient prognosis (32). These insights highlight the role of personalized, gene-targeted therapies in managing prostate cancer. However, extracellular vesicles from the blood sample of case 4 showed that AR-V7 was of the wild type, indicating that the disease’s rapid progression is not related to this variant.

The safety profile and adverse drug reactions are critical in personalized therapy. The CHART study revealed that severe adverse events of grade 3 or higher occurred in 28% of patients treated with rezvilutamide and 21% treated with bicalutamide (10). Such serious adverse events typically included hypertension, hypertriglyceridemia, and weight gain. From the four cases examined, two patients encountered grade 1 adverse reactions, characterized by mild swelling. Given the mildness of the symptoms, treatment continuation was deemed appropriate. The swelling subsided without further complications, indicating the drugs’ relative safety.

## 4 Conclusion

Rezvilutamide, a novel antiandrogen developed in China, shows promise in treating metastatic hormone-sensitive prostate cancer (mHSPC). As more data from clinical trials emerge, the use of new antiandrogens could widen in prostate cancer care, with genetic testing aiding treatment optimization. However, the current study’s small sample size limits the ability to generalize rezvilutamide’s efficacy and safety. Additionally, the short follow-up period hampers our understanding of its long-term effects and potential side effects. Future studies should involve larger cohorts and prolonged observation to thoroughly assess rezvilutamide’s enduring therapeutic value and safety. Gathering extensive real-world clinical data is crucial to confirm its role in mHSPC treatment.

## Data availability statement

The original contributions presented in the study are included in the article/supplementary material. Further inquiries can be directed to the corresponding author.

## Ethics statement

Written informed consent was obtained from the individual(s) for the publication of any potentially identifiable images or data included in this article.

## Author contributions

CZ: Writing – review & editing, Writing – original draft, Software, Methodology, Investigation, Formal analysis, Data curation, Conceptualization. JR: Writing – review & editing, Writing – original draft, Investigation, Data curation. YK: Writing – review & editing, Supervision, Conceptualization. DC: Writing – review & editing, Supervision, Data curation, Conceptualization.

## References

[1] Keam SJ. Rezvilutamide: first approval. Drugs. (2023) 83:189–93. doi: 10.1007/s40265-022-01831-y 36630077

[2] Zhu Y Mo M Wei Y Wu J Pan J Freedland SJ. Epidemiology and genomics of prostate cancer in Asian men. Nat Rev Urol. (2021) 18:282–301. doi: 10.1038/s41585-021-00442-8 33692499

[3] Desai K McManus JM Sharifi N. Hormonal therapy for prostate cancer. Endocr Rev. (2021) 42:354–73. doi: 10.1210/endrev/bnab002 PMC8152444 33480983

[4] Fizazi K Tran N Fein L Nobuaki Matsubara N Rodriguez-Antolin A Alekseev BY. Abiraterone acetate plus prednisone in patients with newly diagnosed high-risk metastatic castration-sensitive prostate cancer (LATITUDE): final overall survival analysis of a randomised, double-blind, phase 3 trial. Lancet Oncol. (2019) 20:686–700. doi: 10.1016/S1470-2045(19)30082-8 30987939

[5] Fizazi K Tran N Fein L Matsubara N Rodriguez-Antolin A Alekseev BY. Abiraterone plus prednisone in metastatic, castration-sensitive prostate cancer. N Engl J Med. (2017) 377:352–60. doi: 10.1056/NEJMoa1704174 28578607

[6] Chi KN Agarwal N Bjartell A Chung BH Pereira de Santana Gomes AJ Given R. Apalutamide for metastatic, castration-sensitive prostate cancer. N Engl J Med. (2019) 381:13–24. doi: 10.1056/NEJMoa1903307 31150574

[7] Sweeney CJ Martin AJ Stockler MR Begbie S Cheung L Chi KN. Testosterone suppression plus enzalutamide versus testosterone suppression plus standard antiandrogen therapy for metastatic hormone-sensitive prostate cancer (ENZAMET): an international, open-label, randomised, phase 3 trial. Lancet Oncol. (2023) 24:323–34. doi: 10.1016/S1470-2045(23)00063-3 36990608

[8] Smith MR Hussain M Saad F Fizazi K Sternberg CN Crawford ED. Darolutamide and survival in metastatic, hormone-sensitive prostate cancer. N Engl J Med. (2022) 386:1132–42. doi: 10.1056/NEJMoa2119115 PMC9844551 35179323

[9] Hussain M Tombal B Saad F. Darolutamide plus androgen-deprivation therapy and docetaxel in metastatic hormone-sensitive prostate cancer by disease volume and risk subgroups in the phase III ARASENS trial. J Clin Oncol. (2023) 41:3595–607. doi: 10.1200/JCO.23.00041 36795843

[10] Gu W Han W Luo H Zhou F He D Ma L. Rezvilutamide versus bicalutamide in combination with androgen-deprivation therapy in patients with high-volume, metastatic, hormone-sensitive prostate cancer (CHART): a randomised, open-label, phase 3 trial. Lancet Oncol. (2022) 23:1249–60. doi: 10.1016/S1470-2045(22)00507-1 36075260

[11] Lin T Chen Y Wu Y Chen S Li X Lin Y. Risk factors for progression to castration-resistant prostate cancer in metastatic prostate cancer patients. J Cancer. (2019) 10:5608–13. doi: 10.7150/jca.30731 PMC6775699 31632505

[12] Huang S Bao B Wu M Choueiri T Goggins W Huang C. Impact of prostate-specific antigen (PSA) nadir and time to PSA nadir on disease progression in prostate cancer treated with androgen-deprivation therapy. Prostate. (2011) 71:1189–97. doi: 10.1002/pros.21334 21656829

[13] Chowdhury S Bjartell A Agarwal N Chung BH Given RW Pereira de Santana Gomes AJ. Deep, rapid, and durable prostate-specific antigen decline with apalutamide plus androgen deprivation therapy is associated with longer survival and improved clinical outcomes in TITAN patients with metastatic castration-sensitive prostate cancer. Ann Oncol. (2023) 34:477–85. doi: 10.1016/j.annonc.2023.02.009 36858151

[14] Matsubara N Chi KN Özgüroğlu M Rodriguez-Antolin A Feyerabend S Fein L. Correlation of prostate-specific antigen kinetics with overall survival and radiological progression-free survival in metastatic castration-sensitive prostate cancer treated with abiraterone acetate plus prednisone or placebos added to androgen deprivation therapy: *post hoc* analysis of phase 3 LATITUDE study. Eur Urol. (2020) 77:494–500. doi: 10.1016/j.eururo.2019.11.021 31843335

[15] Harshman LC Chen Y Liu G Carducci MA Jarrard D Dreicer R. Seven-month prostate-specific antigen is prognostic in metastatic hormone-sensitive prostate cancer treated with androgen deprivation with or without docetaxel. J Clin Oncol. (2018) 36:376–82. doi: 10.1200/JCO.2017.75.3921 PMC5805480 29261442

[16] Pilon D Durkin M Rossi C. (2021), S29. Presented at AMCP Nexus; October 18-21.

[17] Qin X Ji D Gu W Han W Luo H Du C. Activity and safety of SHR3680, a novel antiandrogen, in patients with metastatic castration-resistant prostate cancer: a phase I/II trial. BMC Med. (2022) 20:84–93. doi: 10.1186/s12916-022-02263-x 35241087 PMC8895828

[18] Yamamoto S Sakamoto S Xu M Tamura T Otsuka K Sato K. Testosterone reduction of ≥ 480 ng/dL predicts favorable prognosis of Japanese men with advanced prostate cancer treated with androgen-deprivation therapy. Clin Genitourin Cancer. (2017) 15:e1107–15. doi: 10.1016/j.clgc.2017.07.023 28882738

[19] Klotz L O'Callaghan C Ding K Toren P Dearnaley D Higano CS. Nadir testosterone within first year of androgen-deprivation therapy (ADT) predicts for time to castration-resistant progression: a secondary analysis of the PR-7 trial of intermittent versus continuous ADT. J Clin Oncol. (2015) 33:1151–6. doi: 10.1200/JCO.2014.58.2973 PMC4372851 25732157

[20] Wang Y Dai B Ye D. Serum testosterone level predicts the effective time of androgen deprivation therapy in metastatic prostate cancer patients. Asian J Androl. (2017) 19:178–83. doi: 10.4103/1008-682X.174856 PMC5312215 26975487

[21] Tafuri A Cerruto MA Antonelli A. Neoadjuvant strategies before radical prostatectomy for high risk prostate cancer in the era of new hormonal agents. Curr Drug Targets. (2021) 22:68–76. doi: 10.2174/1389450121666200621194409 32564752

[22] Attard G Murphy L Clarke NW Cross W Jones RJ Parker CC. Abiraterone acetate and prednisolone with or without enzalutamide for high-risk non-metastatic prostate cancer: a meta-analysis of primary results from two randomised controlled phase 3 trials of the STAMPEDE platform protocol. Lancet. (2022) 399:447–60. doi: 10.1016/S0140-6736(21)02437-5 PMC8811484 34953525

[23] Robinson D Van Allen EM Wu YM Schultz N Lonigro RJ Mosquera JM. Integrative clinical genomics of advanced prostate cancer. Cell. (2015) 161:1215–28. doi: 10.1016/j.cell.2015.05.001 PMC4484602 26000489

[24] Abida W Cyrta J Heller G. Genomic correlates of clinical outcome in advanced prostate cancer. Proc Natl Acad Sci. (2019) 116:11428–36. doi: 10.1073/pnas.1902651116 PMC6561293 31061129

[25] van Dessel LF van Riet J Smits M Zhu Y Hamberg P van der Heijden MS. The genomic landscape of metastatic castration-resistant prostate cancers reveals multiple distinct genotypes with potential clinical impact. Nat Commun. (2019) 10:5251. doi: 10.1038/s41467-019-13084-7 31748536 PMC6868175

[26] Chi KN Rathkopf DE Smith MR Efstathiou E Attard G Olmos D. Phase 3 MAGNITUDE study: First results of niraparib (NIRA) with abiraterone acetate and prednisone (AAP) as first-line therapy in patients (pts) with metastatic castration-resistant prostate cancer (mCRPC) with and without homologous recombination repair (HRR) gene alterations. J Clin Oncol. (2022) 40:12. doi: 10.1200/JCO.2022.40.6_suppl.012 34752147

[27] Le DT Durham JN Smith KN Wang H Bartlett BR Aulakh LK. Mismatch repair deficiency predicts response of solid tumors to PD-1 blockade. Science. (2017) 357:409–13. doi: 10.1126/science.aan6733 PMC5576142 28596308

[28] Antonarakis ES Velho PI Fu W Wang H Agarwal N Santos VS. CDK12-altered prostate cancer: Clinical features and therapeutic outcomes to standard systemic therapies, poly (ADP-Ribose) polymerase inhibitors, and PD-1 inhibitors. JCO Precis Oncol. (2020) 4):370–81. doi: 10.1200/PO.19.00399 PMC7252221 32462107

[29] Ditch S Paull TT. The ATM protein kinase and cellular redox signaling: beyond the DNA damage response. Trends Biochem Sci. (2012) 37:15–22. doi: 10.1016/j.tibs.2011.10.002 22079189 PMC3259275

[30] Matsubara N de Bono J Olmos D Procopio G Kawakami S Ürün Y. Olaparib efficacy in patients with metastatic castration-resistant prostate cancer and BRCA1, BRCA2, or ATM alterations identified by testing circulating tumor DNA. Clin Cancer Res. (2023) 29:92–9. doi: 10.1158/1078-0432.CCR-21-3577 PMC9811154 36318705

[31] Sun F Chen H Li W Yang X Wang X Jiang R. Androgen receptor splice variant AR3 promotes prostate cancer via modulating expression of autocrine/paracrine factors. J Biol Chem. (2014) 289:1529–39. doi: 10.1074/jbc.M113.492140 PMC3894334 24297183

[32] Antonarakis ES Lu C Wang H Luber B Nakazawa M Roeser JC. AR-V7 and resistance to enzalutamide and abiraterone in prostate cancer. N Engl J Med. (2014) 371:1028–38. doi: 10.1056/NEJMoa1315815 PMC4201502 25184630

